# A Novel Biosurfactant-Based Oil Spill Response Dispersant for Efficient Application under Temperate and Arctic Conditions

**DOI:** 10.1021/acsomega.3c08429

**Published:** 2024-02-15

**Authors:** Umer Farooq, Ariadna Szczybelski, Frederico Castelo Ferreira, Nuno Torres Faria, Roman Netzer

**Affiliations:** † Department of Petroleum, SINTEF Industry, 7465 Trondheim, Norway; ‡ Norwegian College of Fishery Science, The Arctic University of Norway, 9037 Tromsø, Norway; § Institute for Bioengineering and Biosciences and Department of Bioengineering, Instituto Superior Técnico, Universidade de Lisboa, 1049-001 Lisbon, Portugal; ∥ Associate Laboratory i4HB—Institute for Health and Bioeconomy, Instituto Superior Técnico, Universidade de Lisboa, 1049-001 Lisbon, Portugal; ⊥ Department of Aquaculture, SINTEF Ocean, 7465 Trondheim, Norway

## Abstract

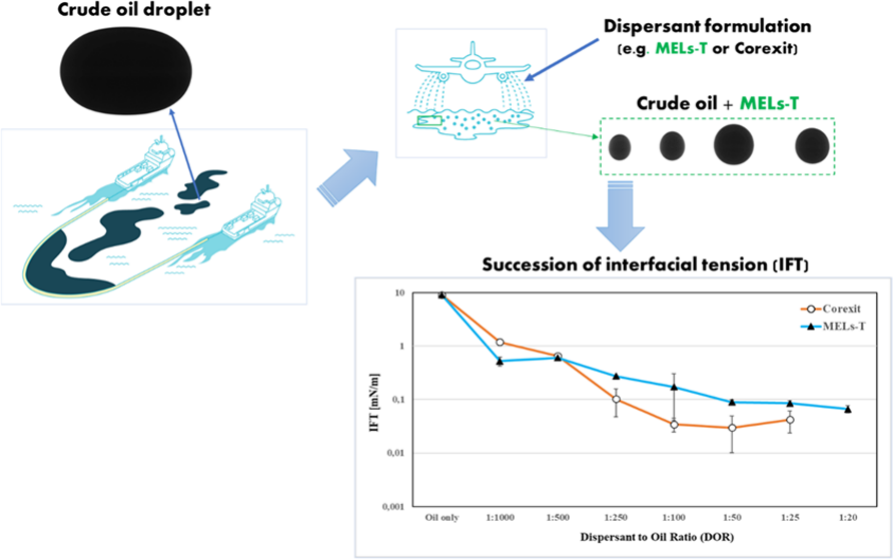

Synthetic oil spill dispersants have become essential in offshore oil spill response strategies. However, their use raises significant concerns regarding toxicity to phyto- and zooplankton and other marine organisms, especially in isolated and vulnerable areas such as the Arctic and shorelines. Sustainable alternatives may be developed by replacing the major active components of commercial dispersants with their natural counterparts. During this study, interfacial properties of different types of glycolipid-based biosurfactants (rhamnolipids, mannosylerythritol lipids, and trehalose lipids) were explored in a crude oil–seawater system. The best-performing biosurfactant was further mixed with different nontoxic components of Corexit 9500A, and the interfacial properties of the most promising dispersant blend were further explored with various types of crude oils, weathered oil, bunker, and diesel fuel in natural seawater. Our findings indicate that the most efficient dispersant formulation was achieved when mannosylerythritol lipids (MELs) were mixed with Tween 80 (T). The MELs–T dispersant blend significantly reduced the interfacial tension (IFT) of various crude oils in seawater with results comparable to those obtained with Corexit 9500A. Importantly, no leaching or desorption of MELs–T components from the crude oil–water interface was observed. Furthermore, for weathered and more viscous asphaltenic bunker fuel oil, IFT results with the MELs–T dispersant blend surpassed those obtained with Corexit 9500A. This dispersant blend also demonstrated effectiveness at different dosages (dispersant-to-oil ratio (DOR)) and under various temperature conditions. The efficacy of the MELs–T dispersant was further confirmed by standard baffled flask tests (BFTs) and Mackay–Nadeau–Steelman (MNS) tests. Overall, our study provides promising data for the development of effective biobased dispersants, particularly in the context of petroleum exploitation in subsea resources and transportation in the Arctic.

## 1 Introduction

The spilling of crude oil into the marine environment has become more frequent over the past few decades as offshore oil exploration and marine transportation have increased.^1−3^ In 2010, the Deepwater Horizon oil spill polluted the Gulf of Mexico with over 210 million gallons of oil.^4,5^ Oil tankers carry millions of gallons of crude oils, posing a significant threat to the marine environment in the event of collision or grounding.^1^ Moreover, oil spills in more vulnerable areas particularly in the Arctic or near the shoreline represent another threat to the environment.^6,7^ One of the most used and accepted methods to clean an oil spill is the employment of dispersants.^8−10^ Chemical dispersants are commonly used as a first step response tool for treating marine oil spills.^11^ During the Deepwater Horizon oil spill, approximately 2.1 million gallons of oil dispersant Corexit 9500A was sprayed onto the oil using aircrafts and ships.^12,13^ Corexit 9500A was also injected subsurface to reduce oil surfacing and subsequent stranding of the oil.^14,15^

The basic principle of the dispersant in oil spill response is to reduce the size of the oil droplets by lowering the interfacial tension (IFT) between the oil and water under the wave action.^15^ This is a process of emulsification, where an effective dispersant must convert the oil slick into discrete droplets (diameter size 1–70 μm) that remain stable to coalescence.^13^ Typically, the smaller the oil droplets, the more efficient the extent of the dispersant, since droplets with a smaller size are more likely to be dispersed by waves.^16^ The oil droplets, which are stabilized by adsorbed surfactant molecules, are then carried below the water surface, where the majority of the oil compounds are subsequently degraded by various microorganisms present in the water column. The fraction of the oil film that is dispersed as droplets into the water column is termed dispersion efficiency (DE). Dispersants such as Corexit 9500A exhibit dispersion efficiencies above 90% for dispersant to oil ratios (DORs) (∼1:20), indicating that they are highly effective at dispersing an oil slick into droplets.

Dispersants currently used in oil spill response are usually a blend of nonionic and anionic surfactants in a solvent base. Surfactants are amphiphilic molecules containing both hydrophobic and hydrophilic regions; therefore, they are soluble in both oil and water phases. The empirical parameter used for the classification of surfactants is the hydrophilic–lipophilic balance (HLB) number. The HLB is defined as
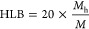
1where *M*_h_ is the molecular weight of the hydrophilic headgroup of the surfactant molecule, and *M* is the molecular mass of the complete molecule, resulting in a scale of 1–20. An HLB value between 1–8 shows a lipophilic surfactant (oil soluble), whereas an HLB value between 12–20 shows a hydrophilic surfactant (water-soluble), each of them promoting the formation of water-in-oil and oil-in-water emulsions, respectively. A surfactant with an HLB between 8–12 may promote either type of emulsion but generally promotes oil-in-water emulsions.^17^

The formulation of Corexit 9500A consists of nonionic sorbitan and polysorbate surfactants, e.g., Tween 80 (HLB 15), Span 80 (HLB 4.3), and Tween 85 (HLB 11) and the anionic surfactant dioctyl sodium sulfosuccinate (DOSS or AOT, HLB 10.9), although the exact mass fraction of each component in the formulation has not been disclosed. A solvent that is commonly used in the formulation to dissolve these surfactants is 1-(2-butoxy-1-methylethoxy) propanol.^18^ There has been a controversial report regarding the toxicity of Corexit 9500A.^15,19−21^ Polysorbates are typically used in foods and cosmetics. On the contrary, the anionic surfactant DOSS in Corexit 9500A is not approved as a food-grade component and considered an irritant to the eyes and skin. DOSS has been reported to slow down the rate of bacterial oxidation of crude oil and to have toxic effects on microalgae and other marine organisms.^19,22,23^ Moreover, DOSS itself does not degrade easily and persists in the ocean longer than the other components of Corexit 9500A.^16^

The increasing environmental concern among the public and the emergence of stricter regulations particularly in Arctic and subarctic regions force the industry to seek alternatives to surfactants produced via petrochemical routes such as biosurfactants (BS). BS, which are derived from microorganisms, are amphiphilic molecules and possess low toxicity and high biodegradability.^24−26^ As the high production cost is the notable limitation to establish BS as an effective surfactant (Onaizi et al.;^27^ Najmi et al.^28^), during the recent years, many studies have been performed where BS were often mixed with inexpensive synthetic surfactants.^16,29−36^ Most BS are either anionic or neutral, whereas those that contain amine groups are cationic. The hydrophobic moiety has long-chain fatty acids, and the hydrophilic moiety can either be a carbohydrate, cyclic peptide, amino acid, phosphate carboxyl acid, or alcohol. BS are generally categorized by their microbial origin and chemical composition, and major classes are (i) glycolipids, (ii) fatty acids/phospholipids/neutral lipids, and (iii) polymeric biosurfactants.^37^ Recently, glycolipids are among the most popular BS, which are characterized by high structural stability and ability to reduce the oil–water IFT significantly.^24,38,39^ Structurally, they are constituted of a fatty acid in combination with a carbohydrate moiety and correspond to a group of compounds that differ by the nature of the lipid and carbohydrate moiety. The best-studied glycolipid BS are rhamnolipids (RLs),^40−45^ trehalolipids (TLs),^46−49^ sophorolipids (SLs),^50−53^ and mannosylerythritol lipids (MELs),^38,54−58^ which contain mono- or disaccharides, combined with long-chain aliphatic acids or hydroxy aliphatic acids.

MELs are glycolipids produced and secreted by *Pseudozyma* spp. with a hydrophilic mannosylerythritol domain with different acetylation degrees and two acylated groups comprising the lipophilic domain made of lipid chains with 8–12 carbons.^58^ MELs are stable at a wide range of temperatures, and as nonionic surfactants, their surface-active properties^56,59^ are stable over a wide pH and ionic strength range. Moreover, in-house empirical experience (results not shown) shows that MELs are stable for more than 12 months, when kept dry, at cooler temperatures (2–8 °C) and protected from light. Due to these properties, MELs are supposed to be suitable for long-term storage. MELs have shown excellent surface-active characteristics, as well as other biochemical functions. Together with their high biodegradability and low toxicity,^60−62^ MELs represent one of the most promising biosurfactant types with high potential for a broad range of industrial applications.^56,58^ In addition, MELs can be produced biotechnologically from lignocellulosic waste material, demonstrating their potential as sustainable alternatives to chemicals produced via petrochemical routes.^54^

While the positive effect of RLs on the biodegradation of organic contaminants is well documented, this has not been reported for MELs, which have a similar amphiphilic structure. Indeed, preliminary studies indicated that MELs can enhance the biodegradation of *n*-alkanes in fresh crude oil and have remarkable surface-active properties, pointing out their high potential for use in environmental applications.^63,64^

In the work reported here, three types of glycolipid surfactants (i.e., RLs, MELs, and TL) were systematically studied for their surface-active properties in a crude oil–seawater system. Among these surfactants, RLs and TLs are generally considered as the most promising candidates for oil remediation and oil spill response.^40,42,65,66^ Yet, only a few studies have tested their dispersant effectiveness in crude oil–seawater systems.^67−69^ Different blends of surfactants were mixed with crude oil, and their IFT was studied against natural seawater. The most promising biosurfactant formulation was further tested with different types of crude oils and at different temperatures, and the dispersing effectiveness was validated against Corexit 9500A. To the best of our knowledge, we here demonstrate for the first time aMELs-based formulation with high potential as environmentally compatible oil spill response agent.^70−72^

## 2 Materials and Methods

### 2.1 Materials

Rhamnolipids (RLs; R90, 90% purity) were purchased from a commercial manufacturer (Merck, Germany), and trehalose lipids (TLs) were kindly provided by Prof. Helen Zhang (The Northern Region Persistent Organic Pollution Control (NRPOP) Laboratory, Faculty of Engineering and Applied Science, Memorial University of Newfoundland, St. John’s, NL, A1B 3 × 5, Canada). Mannosylerythritol lipids (MELs) were produced as described below.

For the preparation of different formulations of dispersants, sorbitan monooleate (Span 80) (CAS# 1338–43–8, Merck—Germany), poly(ethylene glycol) sorbitan monooleate (Tween 80) (CAS# 9005–65–6, Merck—Germany), polyoxyethylenesorbitan trioleate (Tween 85) (CAS# 9005–70–3, Merck—Germany), lighter fuel (combination of hydrocarbons, C_10_–C_13_, *n*-alkanes, iso-alkanes, cyclic, < 2% aromatics), and 2-ethylhexyl acetate (CAS# 103–09–3, Tokyo Chemical Industry Co., Ltd.) were used.

For cleaning of the capillary tube prior to use for IFT measurements, dichloromethane (DCM) (HPLC grade, VWR International AS), toluene (VWR International AS), filtrated natural seawater (SW), and deionized water were used. Deionized water was obtained using a Milli-Q purification system (18.2 MΩ cm at 25 °C).

#### 2.1.1 Synthesis of Mannosylerythritol Lipids (MELs)

MELs were produced by *Moesziomyces antarcticus* using conditions described elsewhere.^73^ Cultivation started with 40 g/L of d-glucose, and after 4 days of cultivation, 20 g/L of waste frying cooking vegetable oil (WFO) was added. After 10 days, cultivated *M. antarcticus* was extracted with ethyl acetate twice and the organic phase was collected and evaporated. The obtained orange gum has a MELs purity of 88–90%, where the main impurities were lipids from unconsumed substrates or produced by the cells. The ratio of the MELs mixture was 68% of diacetylated (MEL-A), 28% of monoacetylated (MEL-B and -C), and 4% of deacetylated (MEL-D). The fatty acid chains ranged from C_8_ to C_12_ with 82% of C_10_. The HLB for the MELs mixture was 8.6.

### 2.2 Crude Oils

In this study, a broad range of crude oils were selected based on their different physical and chemical properties (Table 1). Five different types of oils were selected as representatives of the large number of oils worldwide, covering a large variation in crude oil properties, and are listed below:

- Naphthenic crude oil (Troll B): rich in paraffins and saturated components, low density (or high API gravity), viscosity, and high acid contents;
- Asphaltenic crude oil (Oseberg A): rich in polar resins and asphaltenes, high density (or low API gravity) and low viscosity;
- Waxy crude oil (Norne 2): rich in waxes (higher saturated components > C20), high pour point, low density (or high API gravity), and moderate viscosity;
- IFO 180 Bunker Fuel: intermediate fuel oil (IFO) 180 is the fraction obtained from the petroleum distillation either as a distillate or a residue; and
- Marine Diesel: marine diesel oil (MDO) is a type of fuel oil consisting of a blend of gas oil and heavy fuel oil.

**Table 1:** Physicochemical Properties of Crude Oils, Weathered Oils, and Fuel Oils

oil	TAN	TBN	pour point (°C)	density (g/mL)	viscosity (mPa/s)	asphaltenes (wt %)	waxes (wt %)	IFT (mN/m)
Troll B	1.1	1.3	<−36	0.89	36	0.08	1.8	9.0
Troll B 200 °C+	n.a.	n.a.	n.a.	0.91	n.a.	n.a.	n.a.	n.a
Norne 2	0.3		0	0.88	62	0.18	4.2	11.5
Oseberg A	0.1	2.4	–24	0.90	51	1.19	1.4	15.5
marine diesel	n.a.	n.a.	n.a.	0.85	n.a.	n.a.	n.a.	n.a
IFO 180 Bunker Fuel	n.a.	n.a.	1	0.96	2500	13.8	n.a.	n.a

The total acid number (TAN), total base number (TBN), asphaltene content, wax content, density, and viscosity of the oils were determined according to standard procedures as described by Daling et al.^74^

#### 2.2.1 Evaporation of Crude Oil

Troll B was treated to simulate the evaporation loss of lighter crude oil components during 0.5–1 day of weathering on the sea surface. The evaporation was carried out as a simple one-step distillation at a vapor temperature of 200 °C. The distillation procedure used to simulate evaporation is described by Stiver et al.^75^ The residue was referred to as the weathered fraction 200 °C+.

### 2.3 Seawater (SW)

Seawater was collected either from Trondheimsfjord (Trøndelag, Norway) for IFT measurements and MNS testing or from Logy Bay (Newfoundland, Canada) for baffled flask test (BFT) testing.

For the first case, SW was collected from a depth of 80 m (below thermocline) in a Norwegian fjord (Trondheimsfjord; 63_260N,10_230E), outside the harbor area of Trondheim. The SW is supplied via a pipeline system to SINTEF Ocean laboratories, and the water source is nonpolluted and not influenced by seasonal variations, with a salinity of 34 wt %.^76^

For the second case, SW was collected from a depth of 37 m in Logy Bay (Newfoundland; 47°37′40.7″N, 52°39′41.9″W). The SW is supplied via a pipeline system to the Ocean Sciences Center (Memorial University of Newfoundland, MUN), transported to the Department of Civil Engineering (MUN), and stored at 25 °C prior to use.

### 2.4 Preparation of Biosurfactant-Based Dispersants

Various formulations of dispersants were prepared from different combinations of surfactants and solvents. Lighter fuel (66.7 vol. %) and 2-ethylhexyl acetate (33.3 vol. %) were used as a solvent (S). Initially, a screening of different types of BS was carried out with RLs, TLs, and MELs. These dispersant formulations contained 50 wt % of the corresponding biosurfactant and 50 wt % of solvent. Blends were mixed at 500 rpm at room temperature for 2 h and afterward stored at room temperature.

After analyzing the IFT of different types of BS in the crude oil–SW system, the most effective BS (MELs and RLs) were mixed in different ratios (w/w), either with a surfactant mixture of Tween 80 (T), Tween 85, and Span 80 or only with Tween 80 (T) and solvents (S). Blends were mixed at 500 rpm at room temperature for 2 h and afterward stored at room temperature. Tween 80 (T) was mixed with solvents (S) at a 50:50 wt % ratio at 500 rpm (2 h, room temperature) and used for quality control (S7 in Table 2).

**Table 2:** IFT of Troll B in SW with Different Combinations of Biosurfactants (MELs and RLs), Synthetic Surfactants (i.e., Span 80, Tween 80 (T), and Tween 85), and Solvents^a^

sample	dispersant mixture (wt %)	DOR	IFT 0–100 s (mN/m)	IFT 30 min (mN/m)
S1	50% MELs + 50% solvent	1:5	0.8 ± 0.1	0.8 ± 0.1
S2	48% MELs + 5% span 80 + 5% “T” + 10% Tween 85 + 32% solvent	1:5	0.4 ± 0.1	0.5 ± 0.1
S3	19% MELs + 7% span 80 + 8% “T” + 16% Tween 85 + 50% solvent	1:5	0.07 ± 0.01	0.07 ± 0.01
S4	42% MELs + 28% “T” + 30% solvent	1:5	0.02 ± 0.01	0.04 ± 0.01
S5	42% MELs + 28% “T” + 30% solvent	1:20	0.01 ± 0.01	0.02 ± 0.01
S6	42% RL + 28% “T” + 30% solvent	1:20	0.04 ± 0.01	0.07 ± 0.01
S7	50% “T” + 50% solvent	1:10	0.03 ± 0.01	0.2 ± 0.1

a Solvent contains 66.7 wt % of lighter fuel and 33.3 wt % of 2-ethylhexyl acetate (volume basis). All measurements were made at room temperature. DOR: dispersant-to-oil ratio.

### 2.5 Crude Oil: Biosurfactant-Based Dispersant Premixtures

Crude oils were preheated at 50 °C for 1 h and afterward mixed with different formulations of dispersants at a specific DOR at 500 rpm for 3 h at room temperature. BS formulations were premixed with Troll B in a 1:5 DOR for their initial screening. Thereafter, the chosen BS formulation (MELs + Tween 80), here onward named as MELs–T, was premixed with all different crude oils in a 1:20 DOR, while the control dispersant (T/S) was premixed with Troll B in a 1:10 DOR.

#### 2.5.1 Addition of Corexit 9500A (Corexit)

A commonly used commercial dispersant (Corexit 9500A) was mixed with crude oils and a weathered fraction. Corexit (NALCO Environmental Solutions LLC) contains a mixture of nonionic (48 wt %) and anionic (35 wt %) surfactants, and can be adequate for effective dispersion at breaking wave sea conditions when used at a DOR of 1:100 or less.^14,15^

### 2.6 Interfacial Tension (IFT) Measurements

IFT measurements were performed between crude oil and SW in a spinning drop tensiometer (SVT-20 N with SVTS 20 control and calculation software DataPhysics Instruments GmbH, Filderstadt, Germany) with a heating/refrigerated circulator for temperature control (F12-ED, Julabo GmbH, Seelbach, Germany). Prior to each measurement, the capillary tube was rinsed 3 times with DCM, once with toluene, dried with nitrogen gas, rinsed 3 times with deionized water, dried with nitrogen gas, and then rinsed once with SW. The capillary was carefully filled with SW to ensure the absence of air bubbles. After the capillary was filled with SW, the open side of the fast exchange capillary was closed with a septum held in the septum holder and inserted into the measuring cell.

Crude oil (10–30 μL), premixed with or without dispersant, was injected into the stationary capillary tube with a 1 mL syringe with a long needle. Rotation was then immediately started, and IFT measurements were initiated immediately after preparation of the droplet in the capillary.

The principle of the spinning drop tensiometer is to measure the radius of the oil phase under the high rotational speed by using an optical microscope connected to a computer. The IFT was calculated using the following expression
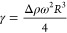
2where γ (mN/m) is the IFT between the oil and water phase; Δρ (g/cm^3^) is the density difference between the crude oil droplet and SW; ω (rad/s) is the angular velocity; and *R* (cm) is the droplet radius. During the first 5 min, IFT was measured after every 5 s, and after this, IFT was recorded after intervals of 30 s. The reproducibility of the experiments was checked by repeating each set of IFT experiments at least twice, and IFT measurements were performed on multiple droplets. The standard deviations were typical, ± 0.2 for high IFT values (1–20 mN/m) and ±0.01 for low IFT values (0.01–0.9 mN/m).

Effectiveness of dispersant mixtures was found to be governed by both the initial oil–water IFT and by the rates of change of oil–water IFT over time (Dynamic IFT).^77^ So, during these studies, both the initial (0–100 s) and dynamic IFTs were measured. Moreover, it is important to use the initial IFT values measured between 0–100 s as the oil droplets need some time to become stable in the capillary tube.^78^ To mimic the leaching effect of the dispersant from the crude oil surface, the IFT values are also calculated after 30–60 min, as the leaching can be measured by changes in IFT.^79^

### 2.7 Dispersibility Tests

Several standardized methods for evaluating the effect of dispersants have been developed over the last decades. The mixing energy input differs in different test methods, so the effectiveness results obtained are strongly influenced by mixing energies applied.

#### 2.7.1 Mackay–Nadeau–Steelman Test

**Figure 1:**
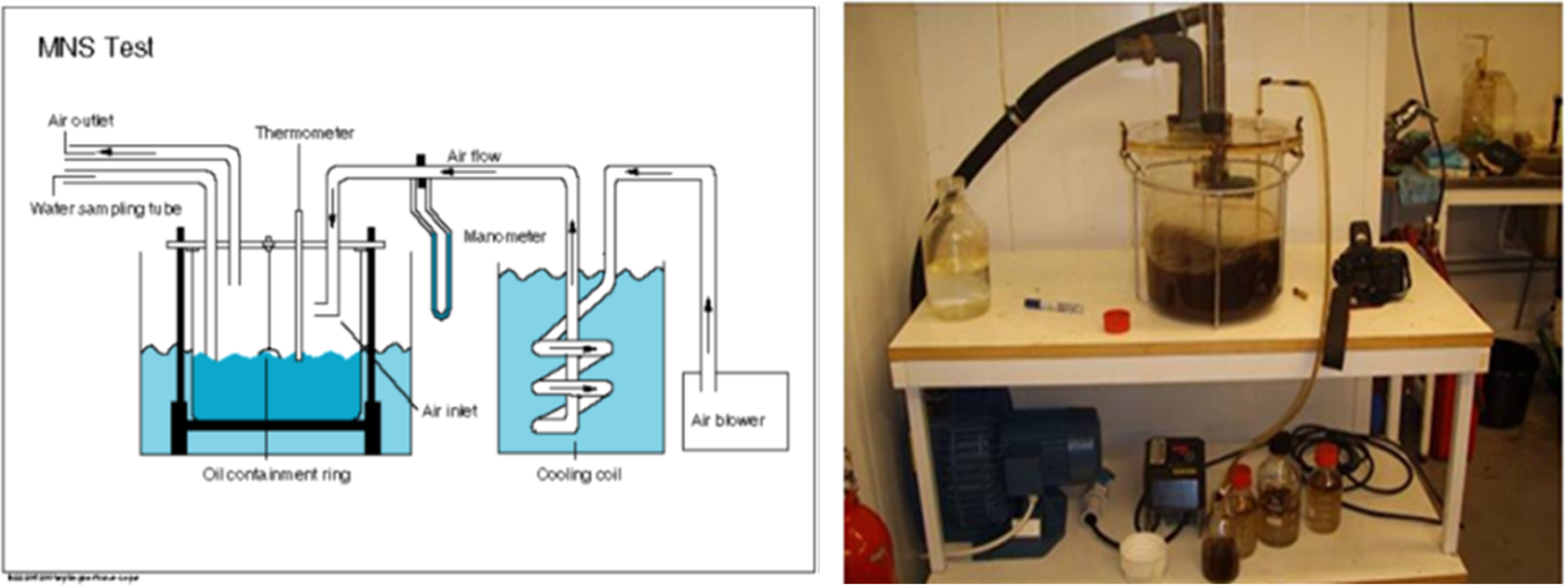
Schematic illustration (left) and image (right) of the Mackay–Nadeau–Steelman (MNS) test. Photograph and schematic illustration credited to Farooq, U.; Brage crude oil–properties and behavior at sea; Sintef Report, 2013.

MNS test^80^ is estimated to represent a medium-to-high sea-state condition (Figure 1). Oil dispersibility was tested with a Corexit 9500A (Corexit) and the most efficient MELs–T dispersant by high energy generating breaking waves during dispersion. The energy input is supplied by blowing air across the oil/water surface to produce a circular wave motion. The MNS test is expensive and time-consuming, and instead of performing replicates on the same oil, Troll B 200 °C + and IFO 180 were included for dispersibility tests. Oil (10 mL) was applied to 6L SW, and then the dispersant was injected on the oil surface at a DOR of 1:25 for Corexit and 1:20 for MELs–T dispersant. The oil was confined in a ring on the SW surface. By removing the ring, the pretreated oil was released and mixed naturally into the SW column during the wave activity. After 5 min of mixing, 500 mL of the oil dispersion was sampled from the system, and oil was extracted using dichloromethane (DCM) liquid–liquid extraction, and extracts were analyzed in an ultraviolet (UV) spectrophotometer at 410 nm for determining the dynamic DE (%). After sampling to determine the dynamic DE, mixing was stopped, and another sample was taken 5 min later to determine the static DE (%).

#### 2.7.2 Baffled Flask Test

BFT was performed according to modifications in the method developed by Sorial et al.^81^ and Zhang et al.^82^ Moreover, in this study, natural SW was used instead of artificial SW. Initially, 120 mL of natural SW was added to a baffled flask, and then, 100 μL of Troll B was carefully added to the SW surface using a 100 μL pipet. Afterward, 4 μL of either Corexit or MELs–T dispersant was added onto the center of the oil slick. The flask was shaken at a rotation speed of 200 rpm on an orbital shaker (ELMI DOS-20L Digital Orbital Shaker 20 mm). After being shaken for 10 min, the flask was left stationary for another 10 min. Then, 2 mL of the mixture was discarded from the stopcock at the bottom of the flask before 30 mL of sample was collected into a 50 mL measuring cylinder. The 30 mL sample was then poured into a separatory funnel after extraction with 5 mL of DCM (HPLC grade) 3 times. Anhydrous sodium sulfate was added into the extract to remove water that may be contained in the solvent. Afterward, the extract was adjusted to a volume of 20 mL for the determination of dispersant efficiency by UV spectrophotometry. The BFT was run in triplicate for the Corexit and MELs–T dispersant. For further details on the preparation of standard crude oil solutions and calculation of DE, see the Supporting Information.

## 3 Results and Discussion

The physicochemical characterization of crude oils, summarized in Table 1, shows that the Oseberg A oil is the most asphaltenic crude oil, containing relatively high concentrations of asphaltenes and basic contents. Troll B is a naphthenic type of crude oil containing a relatively high concentration of acid contents (i.e., TAN > 1 wt %). Norne 2 crude oil is a type of waxy crude oil where wax contents are higher than 4 wt %, while the IFO 180 and marine diesel oils are high-density bunker fuel oil and distillate fuel oil, respectively.

### 3.1 IFT Measurements of Different Types of Glycolipids and BS-Based Formulations

**Figure 2:**
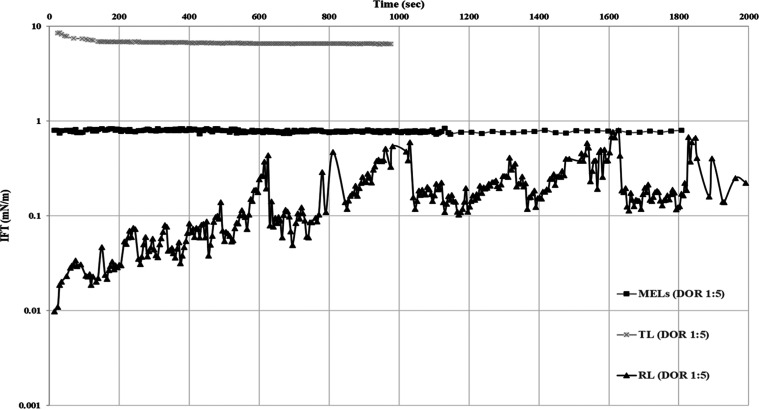
Dynamic IFT of Troll B crude oil in SW with different types of BS (MELs, TLs, and RLs). BS formulations were premixed with Troll B crude oil at a DOR of 1:5 and contained 50 wt % of corresponding BS and 50 wt % of solvent.

The effectiveness of different types of glycolipids was quantified by the magnitude of IFT reduction in the oil/water system. Initially, the IFT of different glycolipids BS was tested with the Troll B crude oil/SW system. The results showed that trehalose lipids (TLs) did not cause any significant reduction in oil/water IFT (Figure 2) and that IFT was reduced to 6 mN/m. This result is in agreement with a previous study reporting that the IFT of TLs against hexadecane/water interface was reduced to 5 mN/m.^47^

On the contrary, rhamnolipids (RLs) caused an immediate and significant drop in IFT of Troll B crude oil, which was initially reduced to 0.01 ± 0.01 mN/m. Afterward, the IFT started to increase and stabilized at an average value of 0.8 ± 0.1 mN/m after 30 min. This typical time-dependent behavior of RLs is attributed to the initial migration of surface-active components to the interface from which RL molecules started to desorb (leaching) into the water phase, resulting in a significant IFT increase (i.e., from 0.01 ± 0.01 to 0.7 ± 0.1 mN/m). The time-dependent behavior of RLs at the oil/water interface might be due to its molecular structure, which makes the packing more difficult at the interface. Generally, the main components of RLs are the hydrophilic head groups, which are monorhamnose (one hydrophilic headgroup) and dirhamnose (two hydrophilic headgroup), and the hydrophobic tail made by fatty acids of specific length (C_10_ or C_12_) and degree of saturation (one or two double bonds). Dirhamnose RLs are bulkier than their monorhamnose counterparts, making their packing much difficult at the oil/water interface, and as soon as the molecules migrate toward the interface (IFT reduction), dirhamnose RLs tend to dominate over monorhamnose RLs and the molecule starts to desorb (increase in IFT).^48^ The dynamic IFT behavior of RLs was also observed by Wu et al. against the decane/water, diesel/water, and toluene/water systems, and IFT was decreased to 20, 28, and 19 mN/m, respectively.^45^ On the contrary, in the study reported by Shreve and Makula, a purified RLs mixture exhibited a minimum IFT value of 0.005 mN/m against hexane.^43^ This showed that the method of preparation, separation, and purification played an important role in the interfacial properties of RLs.

MELs immediately reduced the IFT of Troll B crude oil to 0.8 ± 0.1 mN/m and stabilized it afterward for the entire monitoring period (Figure 2). Our preliminary studies also showed that the IFT of crude oil was reduced by MELs and remained constant over a period of 48 h, with no leaching of surface-active components from the interface (data not shown). Acetyl groups in the hydrophilic sugar moiety play a key role in the interfacial properties, self-assembling, and biochemical properties of MELs.^83^ The HLB of MELs is around 8.6, thus they are more soluble in oil than in the water phase. The MELs molecule has fatty acid chains of C_8_–C_12_ tails, and it is hydrophobic in nature. Once MELs reach the oil/water interface, they will have a low tendency to desorb into the water phase, and the dynamic IFT behavior of MELs with crude oil also provides support to this hypothesis. Previous studies of MELs with HLB 8.8 also showed that their IFT against kerosene oil and *n*-tetradecane decreased to 0.1 mN/m and 2 mN/m, respectively.^55,84^

After screening for the effective and efficient interfacially active BS at crude oil/SW interface, MELs and RLs were mixed with nontoxic and benign components of Corexit (i.e., Span 80, Tween 80 (T), and Tween 85). Different formulations of dispersants were prepared (as shown in Table 2) and premixed with Troll B crude oil. IFT was measured against SW, and the mean values are given in Table 2. The results showed that the lowest and almost stable IFT values were achieved with the dispersant mixture containing 42 wt % of MELs and 28 wt % of Tween 80 (70 wt % surfactants +30 wt % solvents; samples S4 and S5 in Table 2). Such a mixture (60:40 wt % ratio MELs/T blend) reduced the IFT to 0.02 ± 0.01 mN/m or 0.01 ± 0.01 mN/m, respectively, at DOR 1:5 or 1:20, and no significant leaching or desorption of surfactants was observed from the interface (i.e., IFT remains almost constant). On the contrary, with the remaining dispersant mixtures of MELs with different synthetic surfactants (samples S2 and S3 in Table 2), the overall IFT values were higher than for samples S4 and S5. Moreover, when RLs were tested with Tween 80 in the same ratio as with MELs (sample S6 in Table 2), IFT values were not only higher but desorption/leaching was also observed from the interface (i.e., IFT increased from 0.04 ± 0.01 to 0.07 ± 0.01 mN/m after 30 min).

Lastly, the testing of Tween 80 alone, i.e., without adding a BS in the formulation (sample S7 in Table 2) with Troll B crude oil, resulted in significant leaching/desorption of the molecule from the interface, and IFT increased from 0.03 ± 0.01 to 0.2 ± 0.1 mN/m within 30 min. The nature of Tween 80 is discussed by several authors which showed that Tween 80 is a largely hydrophilic and water-soluble compound with HLB = 15. The affinity of this compound toward the water phase is due to the three oxyethylene oligomers present in its headgroup. As soon as the Tween 80 (T) migrates to the oil/water interphase, it started to desorb from the interface to the water phase and hence IFT started to increase after a few minutes.

### 3.2 IFT Measurements of MELs–T and Corexit Dispersants against Different Types of Crude Oils

**Figure 3:**
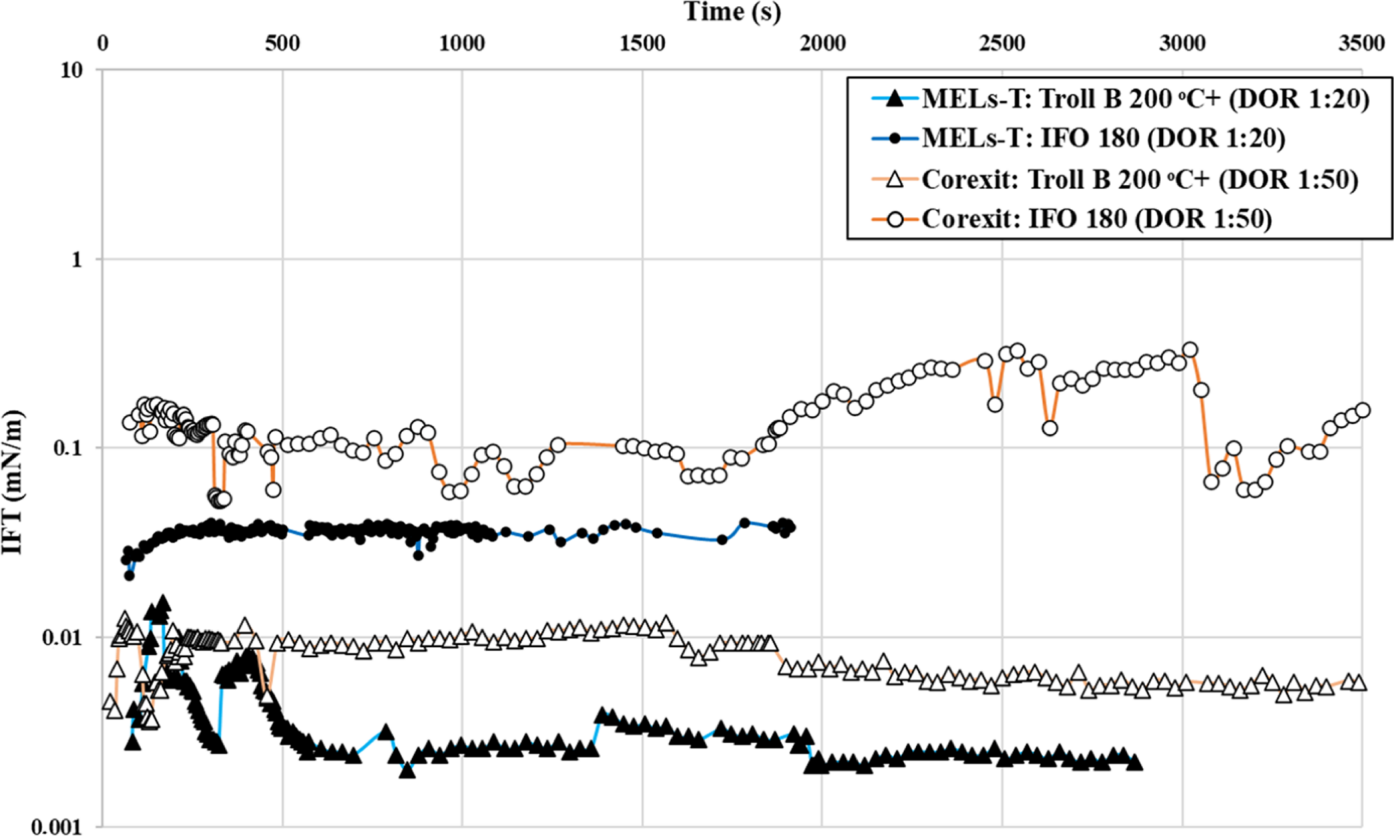
Dynamic IFT of Troll B 200 °C+ and IFO 180 in SW. Oils were premixed with Corexit (DOR 1:50) and MELs–T (DOR 1:20) dispersants. DOR: dispersant-to-oil ratio.

After screening out the different types of BS and respective blends of BS with various nontoxic and benign components of Corexit, it was found that the lowest and most stable IFT was achieved with a dispersant blend containing 42 wt % of MELs and 28 wt % of T (60:40 wt % ratio of MELs/T) at DOR 1:20. Therefore, for subsequent studies with various types of crude oils, weathered oils, and fuel oils, the same dispersant blend of MELs and Tween 80 (MELs–T) was premixed with oils at DOR 1:20. We used Corexit at a 1:50 DOR as a reference since such a dosage yielded the lowest IFT in previous laboratory studies (data not shown). The overall results indicated that with the MELs–T dispersant, the initial (*t* = 0 to *t* = 100 s) and final IFTs (after 60 min) of different types of crude oils varied between 0.004 ± 0.002 and 0.07 ± 0.01 mN/m and between 0.002 ± 0.002 and 0.07 ± 0.01 mN/m, respectively (Table 3). No leaching from the oil/water interface was observed for the MELs–T dispersant blend with any type of crude oils. Furthermore, for all different types of crude oils, the kinetics of the MELs–T dispersant blend were very fast, as the IFT was immediately reduced ∼1000 times. On the contrary, with Corexit, the initial (*t* = 0 to *t* = 100 s) and final IFTs (after 60 min) of different types of crude oils varied between 0.007 ± 0.002 and 0.1 ± 0.1 mN/m and between 0.006 ± 0.002 and 0.2 ± 0.1 mN/m, respectively. Moreover, some leaching/desorption of Corexit was observed from the interface of the IFO 180 bunker fuel oil and SW (Figure 3).

**Table 3:** IFT of Different Types of Crude Oils, Weathered Oils, and Fuel Oils in SW^a^

oil types	IFT (mN/m) 0–100 s MELs–T (DOR 1:20)	IFT (mN/m) 60 min MELs–T (DOR 1:20)	IFT (mN/m) 0–100 s Corexit (DOR 1:50)	IFT (mN/m) 60 min Corexit (DOR 1:50)
Troll B	0.03 ± 0.01	0.02 ± 0.01	0.05 ± 0.01	0.05 ± 0.01
Troll B 200 °C+	0.004 ± 0.002	0.002 ± 0.002	0.01 ± 0.01	0.006 ± 0.002
Norne 2	0.02 ± 0.01	0.008 ± 0.002	0.02 ± 0.01	0.01 ± 0.01
Oseberg A	0.04 ± 0.01	0.02 ± 0.01	0.007 ± 0.002	0.006 ± 0.002
diesel fuel	0.07 ± 0.01	0.07 ± 0.01	0.05 ± 0.01	0.04 ± 0.01
IFO 180 Bunker Fuel	0.03 ± 0.01	0.03 ± 0.01	0.1 ± 0.1	0.2 ± 0.1

a Oils were premixed with Corexit and MELs–T dispersant at DOR of 1:50 and 1:20, respectively. DOR: dispersant-to-oil ratio.

For the naphthenic Troll B crude oil, the IFT was reduced to an average value of 0.03 ± 0.01 mN/m between *t* = 0 and 100 s, and after 1 h, IFT was further reduced to 0.02 ± 0.01 mN/m with the MELs–T dispersant blend. For Troll B crude oil, a higher reduction in IFT was observed with the MELs–T dispersant than with Corexit. Moreover, for the Troll B 200 °C+ weathered fraction, the MELs–T dispersant displayed IFT values even lower than those with fresh Troll B.

For the waxy Norne 2 crude oil, IFT results were very similar between those of the MELs–T dispersant and Corexit. More precisely, the MELs–T dispersant blend and Corexit displayed IFT values ranging from 0.008 ± 0.001 to 0.03 ± 0.01 mN/m and from 0.01 ± 0.01 to 0.02 ± 0.01 mN/m, respectively.

For the asphaltenic Oseberg A crude oil and marine diesel fuel, Corexit displayed slightly lower IFT values than that of the MELs–T dispersant (Table 3).

Finally, for the high-density IFO 180 bunker fuel oil, the MEL–T dispersant revealed much better IFT results than Corexit, with an average IFT of 0.03 ± 0.01 and 0.2 ± 0.1 mN/m, respectively. For high density, weathered, and highly viscous oils, the results indicated that the MELs–T dispersant worked comparatively better than Corexit. These results are very encouraging as, in general, it is more difficult to reduce the IFT of highly asphaltenic, waxy, and weathered oils.

### 3.3 IFT Measurements of MELs–T and Corexit Dispersants against Troll B 200 °C+ Weathered Fraction under Different Temperature Conditions

After evaluating the effectiveness of the MELs–T dispersant with various types of crude oils, Corexit and the MELs–T dispersants were also tested with Troll B 200 °C+ weathered fraction under low (5 °C), temperate (20 °C), and high (60 °C) temperature conditions (Table 4). A temperature of 60 °C is selected because, during the subsea injection of dispersants in the event of subsurface blowouts, the temperature of the released oil from the well exhibits significant variations depending on the reservoir conditions.^85^ It is important to mention that existing oil spill dispersants are mainly developed for marine use from the Arctic to tropic conditions (0–40 °C).^26^ At 5 °C, Corexit displayed IFT values lower than those of the MELs–T dispersant. Still, the IFT was significantly reduced by the MELs–T dispersant, ranging from 0.007 ± 0.002 to 0.009 ± 0.001 mN/m. At 20 °C, both Corexit and the MELs–T dispersant displayed very low IFT values, but IFT results were comparatively better with the MELs–T dispersant, ranging from 0.002 ± 0.001 to 0.004 ± 0.002 mN/m. At 60 °C, Corexit displayed slightly better results than did the MELs–T dispersant. IFT results indicated that the large variation in temperatures did not reduce the effectivity of MELs–T dispersant, and values were in the range of 0.002 ± 0.001 – 0.02 ± 0.01 mN/m.

**Table 4:** IFT Measurement of Troll B 200 °C+ Weathered Fraction in SW under Different Temperature Conditions^a^

oil types	IFT (mN/m) 0–100 s MELs–T (DOR 1:20)	IFT (mN/m) 60 min MELs–T (DOR 1:20)	IFT (mN/m) 0–100 s Corexit (DOR 1:50)	IFT (mN/m) 60 min Corexit (DOR 1:50)
Troll B 200 °C+ (5 °C)	0.007 ± 0.003	0.009 ± 0.002	0.002 ± 0.001	0.001 ± 0.001
Troll B 200 °C+ (20 °C)	0.004 ± 0.002	0.002 ± 0.002	0.01 ± 0.01	0.006 ± 0.002
Troll B 200 °C+ (60 °C)	0.02 ± 0.01	0.01 ± 0.01	0.02 ± 0.01	0.008 ± 0.002

a Troll B 200 °C+ weathered fraction was premixed with Corexit and MELs–T dispersants at a DOR of 1:50 and 1:20, respectively. DOR: dispersant to oil ratio.

### 3.4 IFT Measurements of MELs–T and Corexit Dispersants against Troll B under Different Dosages (DOR)

**Figure 4:**
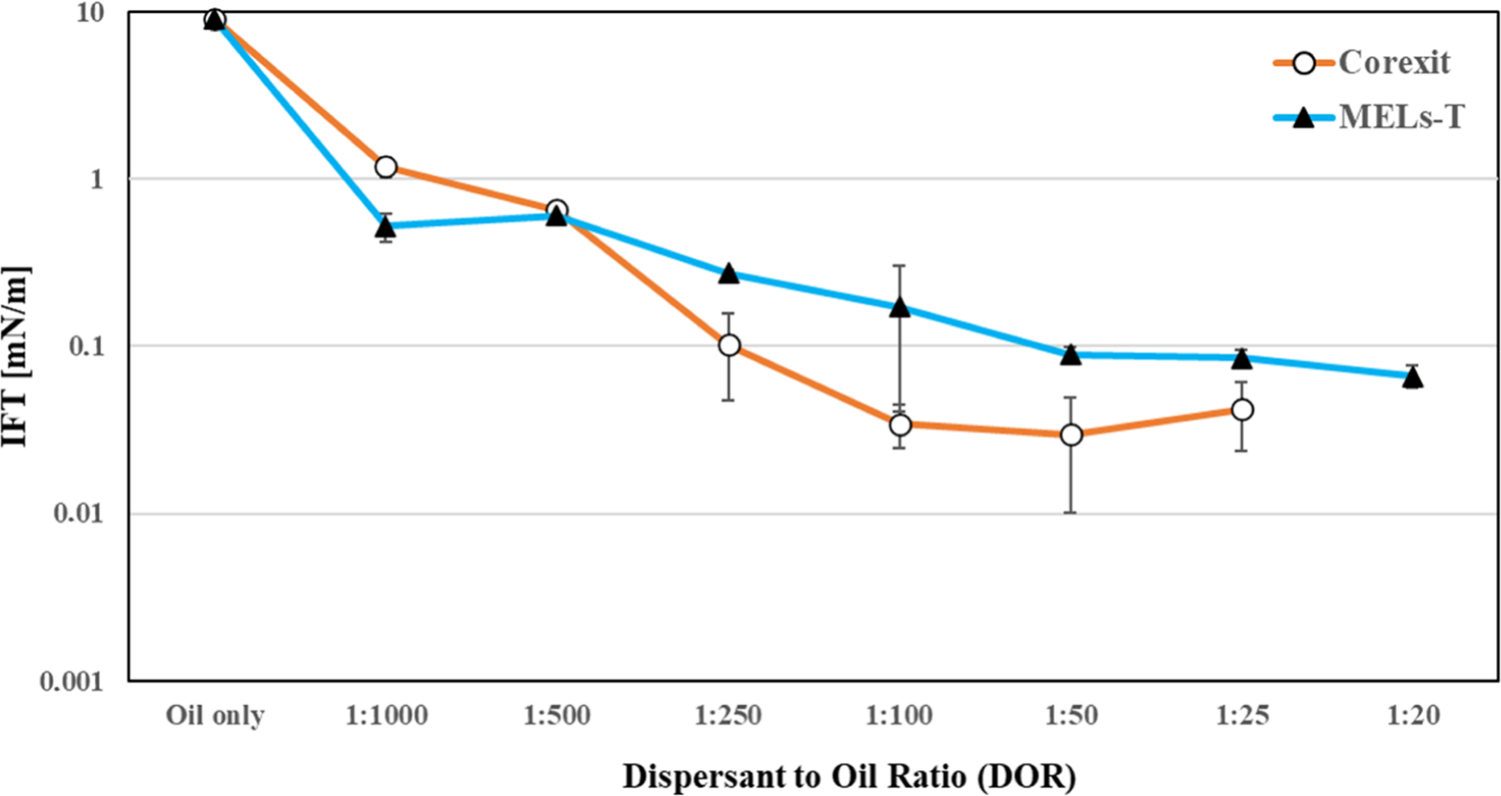
Effect of dispersant-to-oil ratio (DOR) on the IFT of Troll B/SW in MELs–T and Corexit systems.

IFT between Troll B crude oil and SW was also explored as a function of the dispersant dosage or dispersant-to-oil ratio (DOR) (Figure 4). The results clearly indicated that at low DOR (1:1000 and 1:500), the effectiveness of the MELs–T dispersant was better, while at moderate DOR (1:100–1:250) and higher DOR (1:50 and 1:25), Corexit displayed slightly better IFT results than the MELs–T dispersant.

### 3.5 Dispersibility Tests

**Figure 5:**
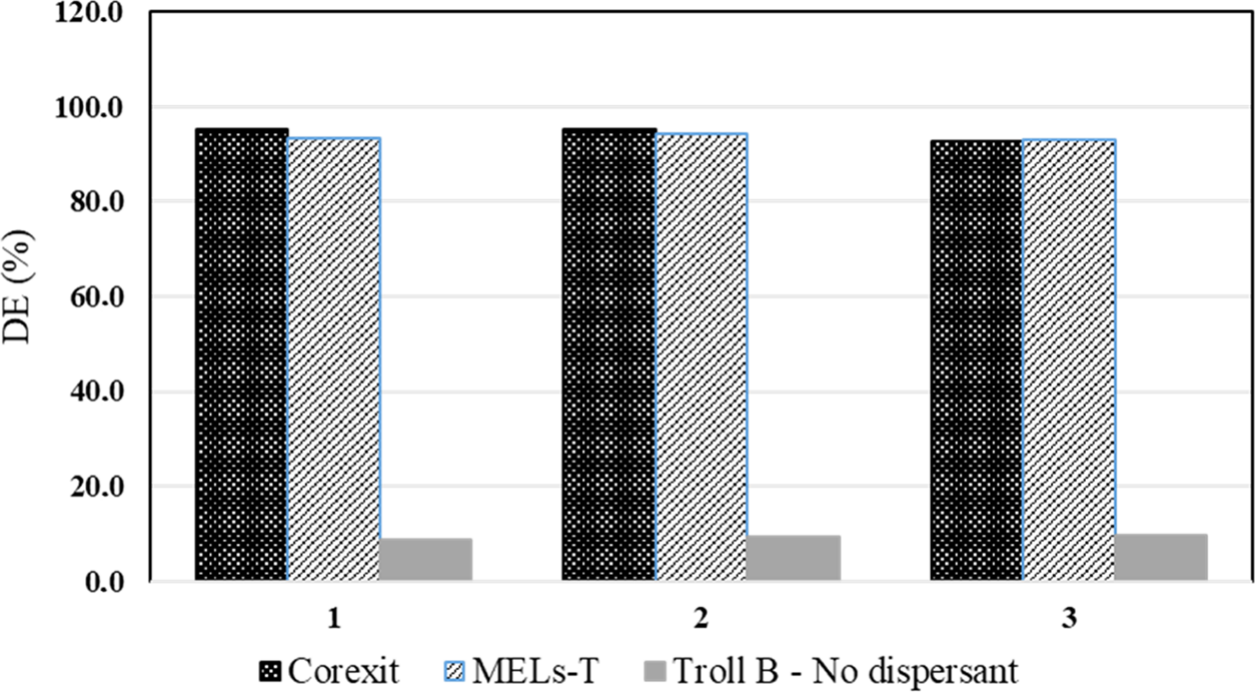
Dispersant effectiveness (DE) for MELs–T (DOR 1:25) and Corexit 9500A (DOR 1:25) in fresh Troll B-oil as obtained in the baffled flask test (BFT) (25 °C). R1–3 are BFT replicates.

After finding the very promising IFT results from the MELs–T dispersant blend, the BFT and MNS tests were performed. Figure 5 shows the DE of Corexit and MELs–T dispersant blend for Troll B crude oil by BFT. Estimated DE values ranged from 93% to 94% or from 93% to 95%, respectively, for MELs–T dispersant blend or Corexit, but for Troll B alone (without dispersant), DE values were below 10%. The BFT results indicated the high dispersibility of Troll B crude oil both by MELs–T blend and Corexit dispersants. The results may, however, be misleading because of the high shear imparted on the oil–water mixtures. The use of alternative dispersibility tests like the MNS test can help to elucidate differences between both dispersants.

For the MNS dispersibility test, weathered oil Troll B 200 °C+ and heavy bunker fuel oil IFO 180 were selected. The MNS test results showed a 100% dynamic DE with both dispersants, while the “blank” sample of Troll B 200 °C+ (without dispersant) also showed DE values of 78%. These results indicate that the mechanical energy of the wave generated in the system for mixing is a major contributor to dynamic DE.

**Figure 6:**
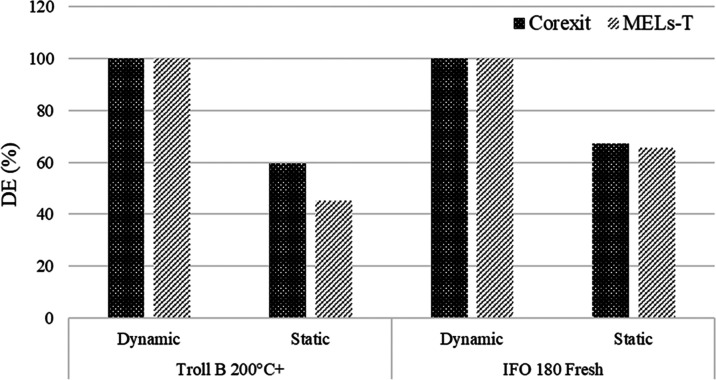
Dispersant effectiveness (DE) for MELs–T (DOR 1:20) and Corexit (DOR 1:25) in Troll B 200 °C+ weathered oil and IFO 180 bunker fuel oil, as obtained in the MNS test at 13 °C. DOR: dispersant-to-oil ratio.

In addition to the dynamic DE, the static DE was determined 5 min after mixing had been stopped. The static DE showed a relatively high dispersibility of bunker fuel IFO 180 (i.e., > 60%), both for MELs–T blend and Corexit dispersants (Figure 6), whereas for Troll B 200 °C+, Corexit had a higher dispersibility (60%) than MELs–T (45%). A different DOR was used for each dispersant based on their effective dosage.

### 3.6 Mechanism for IFT Reduction and Dispersibility by MELs–T Dispersant Blend

The overall results suggested that the MELs–T dispersant blend exhibited excellent interfacial and dispersibility properties, which were comparable with the reference dispersant Corexit. Moreover, for the asphaltenic and high-density crude oils, the IFT results were even better than those obtained with Corexit.

The results can be explained by the aforementioned discussion of the synergy between the “MELs” and “T” molecules. As explained earlier, MELs are hydrophobic, and T is quite hydrophilic and water-soluble. The HLB of MELs and T blend can be calculated by the following expression
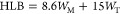
3where *W*_M_ and *W*_T_ are the weight fraction of MELs and T in the blend, respectively. Previous studies showed that an HLB in the range of 9–12 may be optimal for creating an efficient dispersant for oil spill response application. The lowest IFT with Troll B crude oil (0.01 mN/m) was achieved at a 60:40 wt. ratio of MELs and T (42% MELs + 28% T + 30% S), where the HLB is 11.6. Previous studies by Athas et al.^16^ and Jin et al.^31^ observed that the dispersant mixture of lecithin (L) and Tween 80 (T) at the 60:40 wt. ratio with an HLB value of 10.8 exhibited the best emulsification results with crude oil. They found that the L/T blend showed a synergistic effect, but neither L nor T is effective on its own. Moreover, Shah et al.^36^ also studied that the stable oil-in-water emulsion formed at an optimal 60:40 wt.% ratio of lactonic sophorolipid and choline laurate (ionic surfactant). The minimum IFT achieved with the L/T mixture and sophorolipid/choline laurate mixtures, against the crude oil/SW interface, was 0.075 and 1.5 mN/m, respectively. During this study, the IFT results with the MELs–T dispersant blend exhibited much lower values (0.002 ± 0.001 mN/m) and a stable dynamic behavior was observed with a wide range of crude oils (paraffinic, waxy, asphaltenic, marine diesel, and IFO 180) at various temperature conditions. This suggested that the MELs–T molecules pack more closely at the oil/water interface. The close and dense molecular packing at the interface might be due to the favorable interactions between the tail and head group of both MELs and T molecules. The structure of MELs has two fatty acid tails, which are C_8_–C_12_ in length (hydrophobic chains) and a mannosylerythritol head group (hydrophilic head). On the other hand, T has an oleyl tail, which is C_18_ and three hydrophilic oxyethylene head groups.

The synergistic effect of the MELs and T was likely due to the strong hydrophobic van der Waals interaction between the hydrocarbon chains of both molecules. Beside the tail interaction, there is also a favorable interaction between the mannose and oxyethylene head groups of MELs and T molecules, respectively. Athas et al.^16^ suggested that the oxyethylene head group of the T molecule also provides the steric stabilization to the oil droplet by extending into the water phase. We hypothesize that due to the hydrophobic interaction of MELs and T tail groups and steric stabilization of oxyethylene head groups, a very stable interfacial film will be established at the crude oil/SW interface, explaining the significantly lower interfacial tension values obtained and later observed stability for longer period of time, i.e., with no leaching or desorption effect of MELs and T molecules from the interface.

Moreover, IFT results also suggested that the MELs–T dispersant blend demonstrated very low IFT values (0.005 ± 0.003) at 5 and 20 °C with Troll B 200 °C+. IFT decreased with an increase of temperature from 5 °C (0.008 ± 0.002) to 20 °C (0.003 ± 0.002) and then increased slightly upon a further increase of the temperature to 60 °C (0.02 ± 0.01). The change in IFT with temperature may be explained in terms of the complex crude oil composition and temperature-induced structural changes in surfactant molecules. Previous studies demonstrated that IFT does not follow a general trend with an increase in temperature, as it is different for each oil, depending on the crude oil properties and characteristics of surfactant molecules. In most of the cases, IFT and dispersibility of crude oils decreased as the temperature increased from 5 to 20 °C and increased as the temperature increased further.^86−88^ Moreover, temperature is also an important factor in the interaction between head groups of nonionic surfactants, as the interaction between water and hydrophilic group or between oil and lipophilic group changes with temperature. The packing of the T molecules at the interface is also dependent on the temperature. Generally, the critical micelle concentration (CMC) of T decreased with increasing temperature and it slightly increased at higher temperatures.^89,90^

## 4 Conclusions

During this study, an efficient and environmentally friendly dispersant blend was systematically developed for oil spill response applications, which exhibited excellent interfacial properties and dispersibility effectiveness under various conditions. The results were compared with the petroleum-based dispersant Corexit 9500A, which was used as a reference, as it has been applied in large scale in oil spill response in the marine environment. Initially, the IFT of various types of glycolipid surfactants was explored against the crude oil/SW interfaces. MELs and RLs, which lowered the IFT significantly, were further mixed with various environmentally benign and nontoxic components of Corexit 9500A. It was demonstrated that a 60:40 wt ratio of MELs/Tween 80 (T) reduced the IFT of crude oil/SW significantly. Afterward, the interfacial properties of the MELs–T dispersant blend were explored against a wide range of crude oils, weathered, diesel, and heavy fuel oils in SW, and results demonstrated that the MELs–T blend reduced the IFT of oils significantly (0.073–0.002 mN/m). Furthermore, IFT results were also compared with Corexit 9500A and it was found that the MELs–T blend exhibited much better results with weathered and heavy fuel oils. The interfacial properties of both dispersants were also explored under different temperature conditions. BFT and MNS dispersibility tests were performed with the MELs–T dispersant blend and Corexit 9500A against different types of crude oils. BFT results showed that the dispersant efficiency of both dispersants was 93–95% with Troll B crude oil; however, MNS tests displayed slightly better results with Corexit 9500A.

Overall, IFT and dispersibility test results are found to be extremely promising due to their high effectiveness with a wide range of crude oils at different temperature conditions. These results open the window of opportunity for the use of nontoxic and green dispersants in oil spill response applications, particularly under more vulnerable ecosystems, i.e., Arctic conditions and close to shorelines.

The newly developed formulation has the potential to become the first microbial surfactant-based oil spill response agent in the market, replacing conventional solutions such as Corexit and Dasic. It is noteworthy that certain petroleum-based solutions have been discontinued due to their ecotoxicity,^91^ thereby creating an opportunity in the market for more environmentally friendly and efficient solutions, such as the one presented here. However, it is crucial to emphasize that the scaling-up of MELs production is still underway, requiring the design of various production strategies to offer a product at a cost compatible with industry standards.

## Supporting Information Available

Determination of dispersant effectiveness by UV spectrophotometry (PDF)
