# Supplementary material for: A Novel Biosurfactant-Based Oil Spill Response Dispersant for Efficient Application under Temperate and Arctic Conditions
Source: ACS Omega. 2024 Feb 15;9(8):9503–15. doi: 10.1021/acsomega.3c08429 (PMC10905727; doi:10.1021/acsomega.3c08429)
Supplement: Supplementary file 1 — ao3c08429_si_001.pdf [file ao3c08429_si_001.pdf]

A novel biosurfactant based oil spill response dispersant for efficient application under temperate and Arctic conditions

Umer Farooq*[a], Ariadna Szczybelski[b], Frederico Castelo Ferreira[c,d], Nuno Torres Faria[c,d], Roman Netzer[e]

[a]Department of Petroleum, SINTEF Industry, NO-7465 Trondheim, Norway

[b]Norwegian College of Fishery Science, The Arctic University of Norway, Tromsø, Norway.

[c]Institute for Bioengineering and Biosciences and Department of Bioengineering, Instituto Superior Técnico, Universidade de Lisboa, Portugal.

[d] Associate Laboratory i4HB – Institute for Health and Bioeconomy, Instituto Superior Técnico, Universidade de Lisboa, Lisboa, Portugal.

[e]Department of Aquaculture, SINTEF Ocean, NO-7465 Trondheim, Norway

*Corresponding Author

SUPPORTING INFORMATION

Baffled Flask Test. Standard crude oil solutions were prepared for calibrating the ultraviolet spectrophotometer (UVS). Five, 10, 15, 20, and 25 μL Troll B crude oil were added using a syringe to 30 mL DCM (HPLC grade), respectively. These Troll B-DCM solutions were regarded as standard references representing DE of 20, 40, 60, 80, and 100%. UVS was employed to measure the absorbance. The absorbance of the extracts was measured at three wavelengths: 340, 370, and 400 nm, respectively. The area under the absorbance *vs*. wavelength curve between 340 and 400 nm was regarded as the relative concentration of dissolved oil (Chandrasekar et al. 2006). It was determined by the following equation using the trapezoidal rule:

$$Area = \frac{(Abs_{340} + Abs_{370}) \times 30}{2} + \frac{(Abs_{370} + Abs_{400}) \times 30}{2}$$

DE could be calculated as the ratio of the area of dispersed oil to the area of total oil added to the system (equals to the corresponding volume of oil dissolved in DCM). The standard curve was plotted using the value of area to DE. The coefficient of determination was all larger than 0.99.

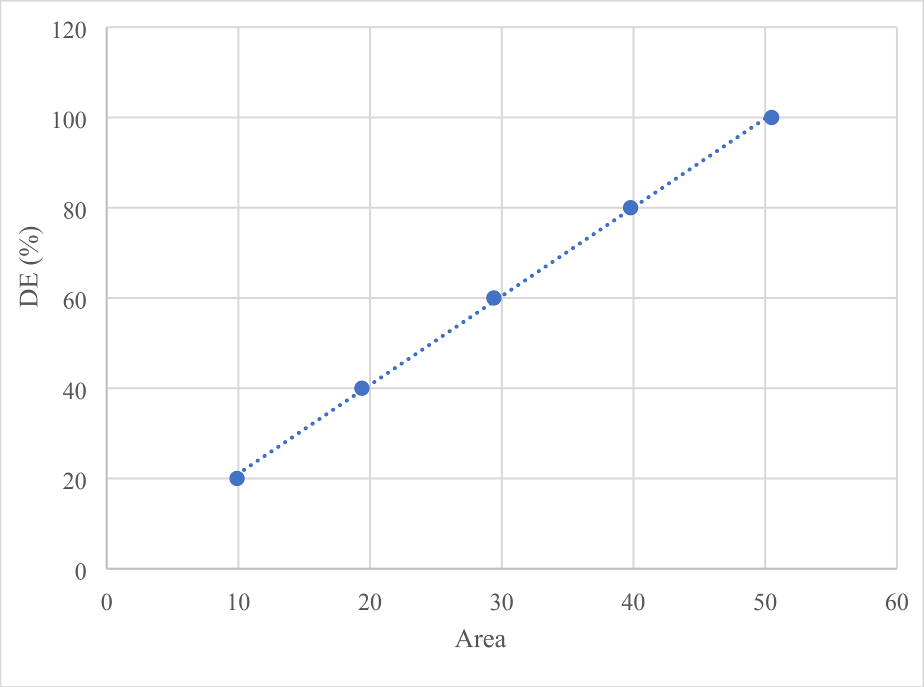

Figure S1: Standard calibration curve for the dispersibility of Troll B crude oil with the MELs formulation. DE: Dispersion effectiveness.

Table S1: Input data for standard calibration curve (Figure S1). DE: Dispersion effectiveness.

**DE (%)**	**Troll B (mg)**	**Abs_{340}**	**Abs_{370}**	**Abs_{400}**	**Area**
20	5.1	0.277	0.145	0.093	9.9
40	10.1	0.544	0.283	0.182	19.38
60	15	0.823	0.431	0.275	29.4
80	20	1.103	0.586	0.377	39.78
100	25	1.405	0.743	0.475	50.49

Table S2: BFT results. DE: Dispersion effectiveness.

Treatment	Replicate	Abs_{340}	Abs_{370}	Abs_{400}	Area	DE (%)
Troll B	1	0.105	0.056	0.035	3.78	8.82
	2	0.116	0.06	0.037	4.10	9.44
	3	0.122	0.059	0.037	4.16	9.56
MELs: Troll B (DOR 1:25)	1	1.302	0.687	0.437	46.70	93.27
	2	1.319	0.691	0.446	47.21	94.27
	3	1.302	0.682	0.439	46.58	93.03
Corexit 9500A: Troll B (DOR 1:25)	1	1.333	0.701	0.446	47.72	95.27
	2	1.329	0.699	0.453	47.70	95.24
	3	1.292	0.681	0.435	46.34	92.56
